# A Digital Microfluidic RT-qPCR Platform for Multiple Detections of Respiratory Pathogens

**DOI:** 10.3390/mi13101650

**Published:** 2022-09-30

**Authors:** Huitao Huang, Kaisong Huang, Yun Sun, Dasheng Luo, Min Wang, Tianlan Chen, Mingzhong Li, Junwei Duan, Liqun Huang, Cheng Dong

**Affiliations:** 1 Zhuhai Center for Disease Control and Prevention, Zhuhai 519087, China; 2 Digifluidic Biotech Ltd., Zhuhai 519000, China; 3 Guangzhou Nansha IT Park Postdoctoral Programme, Guangzhou 511466, China; 4 State Key Laboratory of Analog and Mixed-Signal VLSI, University of Macau, Macao 999078, China; 5 College of Information Science and Technology, Jinan University, Guangzhou 510632, China; 6 School of Intelligent Systems Science and Engineering/JNU-Industry School of Artificial Intelligence, Jinan University, Zhuhai 519000, China

**Keywords:** respiratory pathogens, RT-qPCR, digital microfluidic, on-chip

## Abstract

The coronavirus disease 2019 pandemic has spread worldwide and caused more than six million deaths globally. Therefore, a timely and accurate diagnosis method is of pivotal importance for controlling the dissemination and expansions. Nucleic acid detection by the reverse transcription-polymerase chain reaction (RT-PCR) method generally requires centralized diagnosis laboratories and skilled operators, significantly restricting its use in rural areas and field settings. The digital microfluidic (DMF) technique provides a better option for simultaneous detections of multiple pathogens with fewer specimens and easy operation. In this study, we developed a novel digital microfluidic RT-qPCR platform for multiple detections of respiratory pathogens. This method can simultaneously detect eleven respiratory pathogens, namely, mycoplasma pneumoniae (MP), chlamydophila pneumoniae (CP), streptococcus pneumoniae (SP), human respiratory syncytial virus A (RSVA), human adenovirus (ADV), human coronavirus (HKU1), human coronavirus 229E (HCoV-229E), human metapneumovirus (HMPV), severe acute respiratory syndrome coronavirus 2 (SARS-CoV-2), influenza A virus (FLUA) and influenza B virus (FLUB). The diagnostic performance was evaluated using positive plasmids samples and clinical specimens compared with off-chip individual RT-PCR testing. The results showed that the limit of detections was around 12 to 150 copies per test. The true positive rate, true negative rate, positive predictive value, negative predictive value, and accuracy of DMF on-chip method were 93.33%, 100%, 100%, 99.56%, and 99.85%, respectively, as validated by the off-chip RT-qPCR counterpart. Collectively, this study reported a cost-effective, high sensitivity and specificity on-chip DMF RT-qPCR system for detecting multiple respiratory pathogens, which will greatly contribute to timely and effective clinical management of respiratory infections in medical resource-limited settings.

## 1. Introduction

Acute respiratory diseases caused by viral or bacterial infections have brought a tremendous strain on healthcare systems worldwide due to their high incidence and mortality [1,2]. These infectious agents include 2019 novel coronavirus (SARS-CoV-2), severe acute respiratory syndrome coronavirus (SARS-CoV), Middle East respiratory syndrome coronavirus (MERS-CoV) and influenza A virus (FLUA), and so forth. They all had once put an extreme burden on the pandemic zones’ emergency, hospital and public health systems. Therefore, a fast, sensitive and accurate diagnosis method will significantly contribute to effective disease dissemination surveillance and pandemic expansion prevention [3,4].

Conventional diagnosis strategies include virus culture and serological methods, such as microscopic analysis, colloidal gold immunochromatography, enzyme-linked immunosorbent assay and direct chemiluminescence immunoassay (CLIA), and so forth. However, they are generally time-consuming and labor-intensive or have limited sensitivity and lower specificity issues [4,5,6,7]. In contrast, the molecular diagnosis method, reverse transcription-polymerase chain reaction (RT-PCR), which not only holds a high sensitivity and specificity, but also runs in a rapid speed, has gradually become the primary testing method for detecting and identifying respiratory pathogens worldwide. Nevertheless, this method can only be applied in centralized laboratories due to the tedious procedures and dedicated special instruments requirements [8,9,10,11,12]. In addition, when it comes to multiple detection, the design of multiple primers becomes a huge challenge because the chances of hybridization of primers increase with the number of primers.

Microfluidic technology provides a suitable option for multiple detection with parallel operations. By isolating into different independent micro units, a microsystem can easily perform multiple operations without internal interference. However, most of reported microfluidic devices with multiple detection lean upon channel-based microfluidic technology, whereby auxiliary fluid-handling elements and sophisticated networks of channels are needed to control the motion of an unremitting fluid [13,14]. In contrast, digital microfluidic (DMF) technology uses the electric field to control picoliter- to microliter-sized droplets [15], thus manipulating droplets in an unsurpassed flexible way without complicated networks and auxiliary elements. By virtue of accurate fluid manipulation and low sample volume consumption traits, DMF has made clinical biomedical testing simpler and more accessible in the field setting. Additionally, the required time for amplification and analysis steps can also be reduced due to the microfluidic system’s rapid mass and heat transfer [16]. 

Conventional RT-PCR testing generally involves multiple separated procedures such as nucleic acid extraction, reverse transcription, PCR amplification and data analysis, where an experienced expert is needed to avoid operation mistakes [17,18,19]. Nevertheless, all these biological procedures can be integrated and automated into the DMF chip by designing multiple independent reaction chambers in the DMF system, thus simplifying the operation process. Therefore, DMF technology has recently been widely applied to immunological, biochemical and molecular diagnostics tests [20,21,22]. However, simultaneous detection of more than ten respiratory pathogens by quantitative real-time RT-PCR (RT-qPCR) on the DMF platform is yet to be reported.

**Figure 1:**
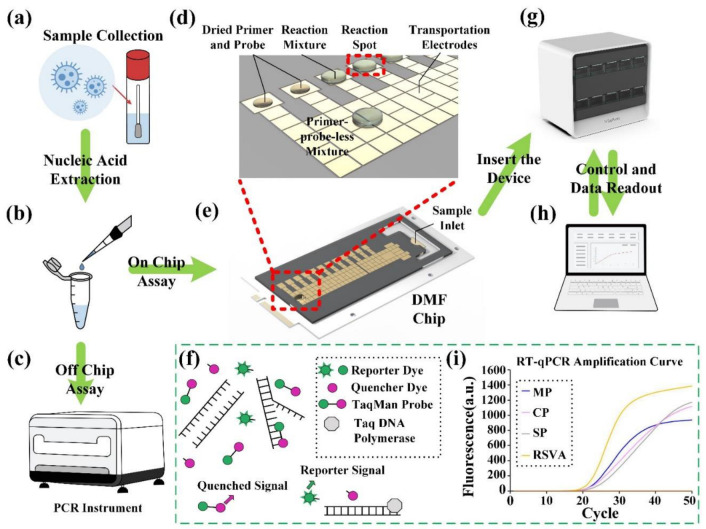
Schematic illustration of the processes of the DMF system for detecting multiple respiratory pathogens using the RT-qPCR method. (**a**) Clinical sample collection. (**b**) Off-chip nucleic acid extraction. (**c**) 3D schematic representation of the PCR thermocycling instrument. (**d**) Detailed illustration of the DMF chip with dried and rehydrated primer probes. (**e**) 3D schematic illustration of the DMF chip. (**f**) Schematic representation of RT-qPCR process. (**g**) The photograph of the DMF nucleic acid detector that is used to drive the DMF chip. (**h**) A custom software on a computer for controlling the device and collecting data. (**i**) Amplification curve obtained from RT-qPCR amplification.

This study sought to develop an eleven-respiratory pathogens detection method based on RT-qPCR and DMF technology. The eleven common respiratory pathogens are Mycoplasma pneumonia (MP), Chlamydophila pneumoniae (CP), Streptococcus pneumonia (SP), human respiratory syncytial virus A (RSVA), human adenovirus (ADV), human coronavirus HKU1 (HCoV-HKU1), human coronavirus 229E (HCoV-229E), human metapneumovirus (HMPV), SARS-CoV-2, FLUA and influenza B virus (FLUB). In our DMF cartridge, they can be detected in a run by using 12 reaction spots on a single DMF chip (Figure 1). The performance characteristics of this DMF device have been evaluated by comparing it with off-chip testing and using clinical sample analysis. Furthermore, due to the pre-programmed configuration, this device can be operated by non-professional individuals and used in field conditions, thus providing a powerful tool for respiratory pathogen detection in medical resource-limited settings. 

## 2. Materials and Methods

### 2.1. Samples

The positive plasmids harboring the targeted gene regions are synthesized by Sangon Biotech (Shanghai) Co., Ltd. (Shanghai, China) (Table 1). In addition, 40 clinical nasal or throat swab specimens (1 SP and HMPV-positive sample, 3 MP-positive samples, 1 SP-positive sample, 3 RSV-positive samples, 3 ADV-positive samples, 1 HMPV-positive sample, 2 SARS-CoV-2-positive samples, 7 FLUA-positive samples, 6 FLUB-positive samples and 13 negative samples from healthy individuals), were collected from 2019 to 2021 in strict accordance with the standard clinical sample collection protocol, with written consent from the patients and the volunteers and ethical approval from Zhuhai CDC ethic committee respectively.

### 2.2. RNA Extraction

The total nucleic acid of clinical specimens is extracted using the RaPure Viral RNA/DNA Kit (Magen, Guangzhou, China) according to the manufacturer’s instructions. Briefly, 250 μL of sample stocks are mixed with 500 μL of Buffer GRP and incubated for 10 min. Then, the lysates are fractionated, and the nucleic acid is subsequently eluted from cationic resin columns in Nuclease Free Water. Finally, the extracted nucleic acid is used for on-chip and off-chip RT-qPCR testing.

### 2.3. Primer and Probe Sequences

The primers and probes sets targeting the matrix gene 1 & 2 of FLUA, the hemagglutinin of FLUB and the ORF1ab and Nucleocapsid gene of SARS-CoV-2 are developed as previously reported [23,24]. The primer–probe sets for the other eight respiratory pathogens are designed using the Primer Premier 5 software according to the corresponding conserved region sequence. All primers and probes are commercially synthesized from Sangon Biotech (Shanghai, China) Co., Ltd. and are dissolved in molecular grade RNAse-free water (Sigma, St. Louis, MO, USA) at 10 μM. The detailed oligonucleotide primers and probes and their sequences are listed in Table 1.

### 2.4. System Configuration

**Figure 2:**
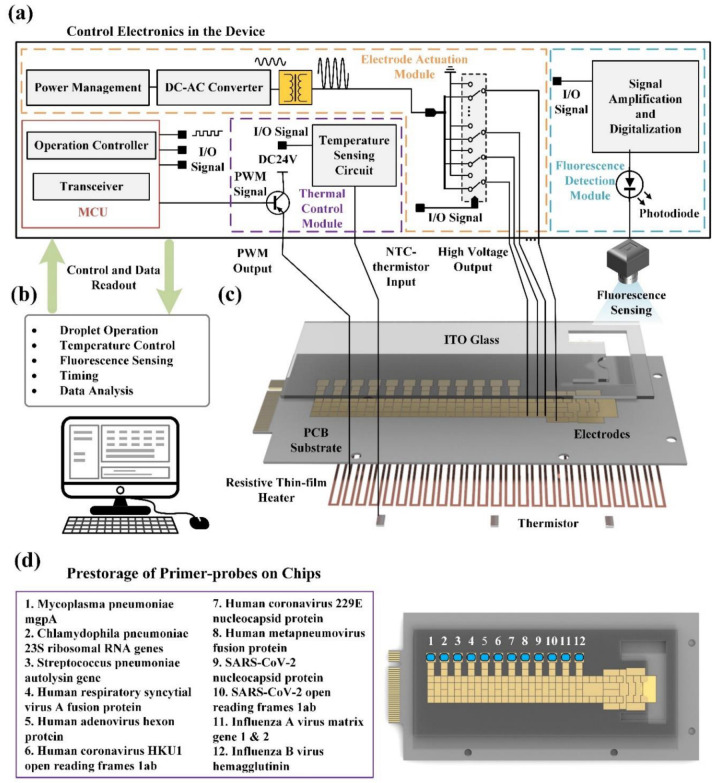
The overview of the DMF system configuration. (**a**) The control electronics in the device; (**b**) The customized and user-programmable software on the PC; (**c**) A 3D explosion diagram of the proposed DMF chip; (**d**) The prestorage of corresponding primer–probe pairs on-chip.

An overview of the proposed DMF system is shown in Figure 2. The DMF RT-qPCR system consists of the DMF chip (Figure 2c), control electronics in the device (Figure 2a), and programmable control software on a personal computer (PC) (Figure 2b). On the DMF chip, the primer–probe solution is spotted at the reaction spots on the bottom plate and dehydrated as in Section 2.6 (Figure 2d).

The control electronics device integrates a microcontroller unit (MCU), an electrode actuation module, a thermal control module and a fluorescence module. The MCU communicates with the PC through a blue tooth data link by a transceiver module, which transmits the real-time data collected by the device and receives commands sent by the PC. The operation controller module controls the rest of the hardware to perform operations according to the commands. Electrode driving potentials are generated by a DCAC converter (direct current to alternating current converter) and amplified by a voltage transformer. The amplified potentials of 260 Vrms are applied for the generation and transportation of the liquid droplets through an array of physical relays controlled by the I/O signal from the MCU. A fluorescence detector is installed on the top of reaction spots of the DMF chip for real-time signal collection, which is then transformed into an electrical signal by a photo-diode. After signal amplification and digitalization, the final fluorescence data are transmitted to the MCU. The thermistor mounted under the PCB is connected to the temperature sensing circuit. A pulse width modulation (PWM) signal is generated according to the proportion integration differentiation (PID) controller with two inputs: the real-time temperature and the target temperature. The PWM signal is connected to the resistive thin-film heater after amplification by a power amplifier for temperature control.

The customized and user-programmable software on the PC enables users to monitor and control the status of the DMF chip in real time through a graphical user interface (GUI). In addition, it provides droplet operation, temperature control, fluorescence sensing, timing, and data analysis capabilities.

### 2.5. RT-qPCR Off-Chip

RT-qPCRs are conducted in a 25 μL reaction mixture containing 5 μL RNA/DNA, 0.5 μL forward primers, 0.5 μL reverse primers, 0.25 μL probes, 5 μL RNAse-free water, 12.5 μL 2× One Step Q Probe Mix and 1.25 μL One Step Q Probe Enzyme Mix (HiScript II One Step qRT-PCR Probe Kit, Vazyme, Nanjing, China). The mixes contain all components necessary for RT-qPCR amplifications except primers, templates and water. Thermal cycling parameters are 15 min at 50 °C, followed by 30 s at 95 °C, and a subsequent 45 cycles of 95 °C for 10 s and 60 °C for 30 s. The AGS4800 quantitative real-time PCR is performed using the quantitative PCR thermocycler instrument (Daan Gene, Guangzhou, China).

### 2.6. Prestorage of Primer-Probes On-Chips

In order to detect multiple items, the 12 primer–probe sets of respiratory pathogens are pre-stored in DMF chips. The prestorage solution consists of 1 μL of 10 μM forward primers, 1 μL of 10 μM Reverse primers, 0.5 μL of 10 μM probes, 2.5 μL of 10% PEG6000 (Sigma, St. Louis, MO, USA) and 20 μL of RNAse-free water (Sigma, St. Louis, MO, USA). The prestorage solution is divided into 2 μL to the corresponding reaction point in DMF chips. The chips with prestorage solution are placed in Shanghai Yiheng electric heating blast drying oven DHG-9075A at 50 °C/20% rh for 15 min. Afterward, the dried chips are taken out for packaging. The DMF chip with pre-stored primer–probe sets is shown in Figure 2.

### 2.7. RT-qPCR On-Chip

All RT-qPCRs on the chip are performed using the DMF nucleic acid detector and HiScript II One Step qRT-PCR Probe Kit (Vazyme, Nanjing, China) following the manufacturer’s instructions. First, the amplification mixture is prepared by using 5 μL RNA, 6.25 μL RNAse-free water, 12.5 μL 2× One Step Q Probe Mix and 1.25 μL One Step Q Probe Enzyme Mix (HiScript II One Step qRT-PCR Probe Kit, Vazyme). Then, the DMF chip with pre-stored primer–probe sets is placed into a metal cartridge. Next, 530 μL of silicone oil is slowly added to the oil inlet of the DMF chip, and 16 μL of the amplification mixture is instilled into the sample inlet of the DMF chip. The cartridge with the DMF chip is inserted into the DMF nucleic acid detector, which automatically completes the pipetting, amplification, and detection procedure. During the pipetting process, 2 μL amplification mixture is transferred to each reaction chamber on the chip. The amplification reaction procedure is the same as the RT-qPCR off-chip in Section 2.5. Cycle 3 to 15 is taken as the baseline period by default, and the threshold value is set at ten times the standard deviation above the mean baseline value. Cycle threshold (Ct) values are calculated according to this standard in subsequent experiments. Forty samples from Section 2.1 are extracted to perform RT-qPCR assay both on-chip and off-chip, as mentioned in Section 2.5 and Section 2.7.

### 2.8. Data Analysis

The coefficient of variance (CV) is calculated based on Ct values. Repeatability (three replicates for one experiment) and reproducibility (three-time experiments) of on-chip RT-qPCRs are shown as CV values.

The screening testing results present as true positive (TP), false positive (FP), true negative (FN) and false negative (TN). The true positive rate (TPR) is calculated as TPR = TP/(TP + FN) × 100%. The true negative rate (TNR) is calculated as TNR = TN/(FP + TN) × 100%. The positive predictive value (PPV) is calculated as PPV = TP/(TP + FP) × 100%. The negative predictive value (NPV) is calculated as NPV = TN/(FN + TN) × 100%. Accuracy (ACC) is calculated as ACC = (TP + TN)/(TP + TN + FP + FN).

Kappa test is performed with SPSS v16.0 software to estimate the degree of consistency between RT-qPCRs on-chip and off-chip. The Kappa statistic is interpreted as follows: <0, as poor; 0–0.20, as slight; 0.21–0.40, as fair; 0.41–0.60, as moderate; 0.61–0.80, as substantial; and 0.81–1.00, as almost perfect agreement [25].

## 3. Results

### 3.1. Optimization of RT-qPCR Reaction

This study aims to detect and differentiate respiratory pathogens using RT-qPCR technology and DMF technology. To verify the amplification performance of each primer–probe set, RT-PCR oligomers are tested with 10 pg/μL of corresponding plasmids using AGS4800 real-time fluorescence quantitative PCR instrument with reference to HiScript II One Step qRT-PCR Probe Kit. For the twelve primer–probe sets, most of the amplification reactions reach the threshold at around 20 cycles, and no false-positive reactions are observed until 45 cycles. The maximum relative fluorescence intensity (RFI) of the twelve primer–probe set amplification reactions is distinct when they reach a plateau. The larger the variance of fluorescence intensity in the plateau phase, the less the accuracy of the fluorescence system is presented. To make the endpoints of the graph relatively concentrated for twelve primer–probe sets on the same chip, the probe concentration is adjusted under the condition that the concentration of forward and reverse primers remain unchanged (Table 2). After adjustment, the maximum RFI of each primer–probe in the plateau phase is about 1.3~1.8. The threshold value is unified and the accuracy of the fluorescence system is improved. In addition, the primers are dehydrated on the chip to reduce the operating time for fluid preparation of testers, leading to improved convenience.

### 3.2. Sensitivity of RT-qPCR On-Chip and Off-Chip

**Figure 3:**
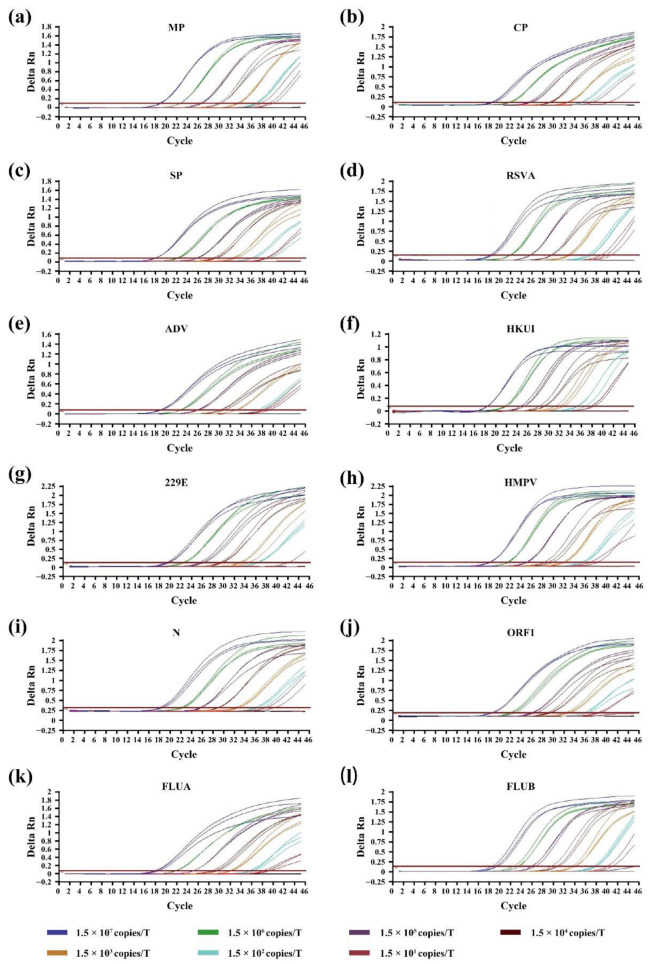
The sensitivity of the microfluidic RT-qPCR assays off-chip. Amplification curves show the detection of pathogens plasmids of (**a**) MP, (**b**) CP, (**c**) SP, (**d**) RSVA, (**e**) ADV, (**f**) HKU1, (**g**) 229E, (**h**) HMPV, (**i**) N, (**j**) ORF1, (**k**) FLUA and (**l**) FLUB.

**Figure 4:**
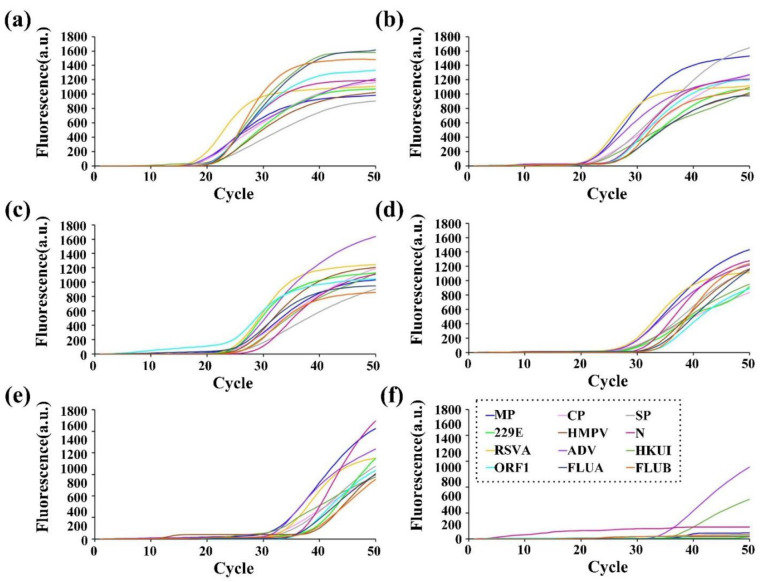
The sensitivity of the microfluidic RT-qPCR assay on-chip. Amplification curves of MP, CP, SP, RSVA, ADV, HKU1, 229E, HMPV, N, ORF1, FLUA and FLUB are shown in detection of (**a**) 1.2 × 10^6^ copies/T, (**b**) 1.2 × 10^5^ copies/T, (**c**) 1.2 × 10^4^ copies/T, (**d**) 1.2 × 10^3^ copies/T, (**e**) 1.2 × 10^2^ copies/T and (**f**) 1.2 × 10^1^ copies/T.

After the final concentration of primers and probes is established, we evaluate the sensitivity of primer–probe sets in detecting different diluted QC plasmids concentrations of 11 respiratory diseases both off-chip and on-chip. It should be noticed that among those 11 respiratory diseases, we have 12 target genes since two gene regions (ORF1ab and N) are selected for SARS-CoV-2 detection. It is first evaluated using plasmids ranging from 1.5 × 10^1^ to 1.5 × 10^7^ copies per test for off-chip testing (Figure 3a–l) and then from 1.2× 10^1^ to 1.2 × 10^6^ copies per test for on-chip testing (Figure 4a–f). There are amplification curves in the concentrations from 1.2 × 10^2^ to 1.2 × 10^6^ copies per test in all 12 detection tunnels (Figure 4a–e), and the amplification efficiency of each reaction is 90 ± 5%. CV values vary from 0.28% to 3.12% at the concentrations from 1.5 × 10^3^ to 1.5 × 10^7^ copies per test off-chip, while CV values range from 0.32% to 4.46% at the concentrations from 1.2 × 10^3^ to 1.2 × 10^6^ copies per test on-chip. The intra-assay variation for repeatability is 0.32% to 3.78%, and the inter-assay variation for reproducibility is 0.43% to 4.46%. Additionally, for each pathogen, over 95% of replicates (≥19 out of 20 replicates) can be detected at each detection limit, suggesting a high sensitivity for the RT-qPCR on the DMF chip. The high linearity of all 12 standard curves off-chip (Table 3) and on-chip (Table 4) also demonstrates the feasibility of using this RT-qPCR assay for the quantification of pathogen nucleic acid in clinical samples. 

### 3.3. Specificity of RT-qPCR On-Chip

The specificity of RT-qPCR on the chip is evaluated using the QC plasmids. Firstly, the negative and different target-positive QC plasmids are employed to test the sensitivity of RT-qPCR assay on the developed DMF system. Each of the twelve different primer–probe sets only shows a positive amplification in its corresponding QC plasmid group, and a negative result in the negative QC and other remaining pathogen QC plasmid groups (Table S1). On the other hand, six QC plasmids harboring the targeted gene regions such as Middle East respiratory syndrome (MERS) and human parainfluenza virus (PIV), which was not incorporated into our system, are also tested to be negative, as shown in No.13 to No.18 (Table S1). No.19 to No.38 are blank control groups adding ddH_2_O instead of plasmids. These results demonstrate the high specificity of our DMF system.

### 3.4. Evaluation of On-Chip RT-qPCR Assays Using Clinical Samples

Thirteen clinical nasal or throat swab samples from healthy controls and twenty-seven samples from patients with the previously confirmed presence of one of the 11 pathogens are used to verify the reliability of our methods in practical use. After RNA extraction in Section 2.2, parallel experiments were performed simultaneously, both on-chip and off-chip (Table S2), for verification purposes, as mentioned in Section 2.5 and Section 2.7. Table 5 summarizes the screening result, with a total of 30 positive testing results and 450 negative testing results confirmed. For detecting these 11 target respiratory pathogens, TPR, TNR, PPV, NPV and ACC measures are utilized for detection performance evaluation. According to the evaluation method in Section 2.8, the results are TPR 93.33%, TNR 100%, PPV 100%, NPV 99.56%, and ACC 99.58%, respectively. The results suggest that the on-chip method can effectively identify 11 pathogens with high accuracy. In addition, the testing results comparison between the two approaches is analyzed using the Kappa coefficient. The Kappa value is 0.963, and the approximate significance (Approx.Siq.) is less than 0.5 (Table 6). These results prove that the RT-PCR testing on-chip and off-chip is highly consistent in clinical practice.

## 4. Discussion

Most respiratory pathogen infections cause similar clinical symptoms characterized by fever, headache, cough, and so forth. Nevertheless, some pathogens are more contagious and can process to be severe acute respiratory syndrome with a high fatality rate. The traditional RT-qPCR detection method needs to configure multiple reaction systems when aiming to detect multiple items, leading to a time-consuming and laborious process. However, a digital microfluidic RT-qPCR chip can integrate numerous qPCR reactions step into the independent spots, thus achieving the simultaneous detection of multiple pathogens. Therefore, in contrast to the traditional RT-qPCR detection method, the DMF RT-qPCR chip provides the advantages of automatic operation and simultaneous detection of more items.

The novelty of this work lies in the implementation of our automated DMF platform and optimized RT-qPCR reaction for simultaneous diagnosis of multiple respiratory pathogens. Compared to conventional diagnosis methods (Table 7), such as bacterial culture, immunofluorescence and enzyme linked immunosorbent assay (ELISA), with which it is generally difficult to reach a balance between high sensitivity, high specificity and rapid diagnosis [26], the digital microfluidic RT-qPCR platform offers a simple operating process, i.e., loading nucleic acid for just one time, as well as high sensitivity and accuracy. While other microfluidic devices with isothermal amplifications (such as loop-mediated isothermal amplification, LAMP for short) generally cannot achieve multiple detection and distinguish pathogenic microorganisms from resident ones [27], our DMF platform with RT-qPCR demonstrates a simultaneous screening of eleven respiratory pathogens. The clinical evaluation illustrates that the PPV is 93.33% and NPV is 100%. Two clinical samples out of thirty were tested to yield a false negative using the off-chip method. We speculate that the decreased sensitivity can be attributed to the fewer nucleic acid template in the reactions. In the RT-qPCR reaction mixture formula, the sample volume for nucleic acid extraction in these two methods is the same. However, the DMF on-chip RT-qPCR only adds 0.4 μL of extracted nucleic acid for each reaction, while the conventional off-chip RT-qPCR method adds 5 μL of extracted nucleic acid instead. This is 12.5 times higher than that of the on-chip way. With a very small sample volume, the DMF on-chip RT-qPCR method can significantly decrease the risk of human contact during sampling. In addition, the use of small sample volumes is also conducive in the case of limited samples in clinical settings.

In conclusion, the digital microfluidic RT-qPCR method designed in this study can rapidly detect multiple common respiratory pathogens with a reduced sample consumption in a fully integrated manner. Additionally, this method shows a comparable amplification performance to the traditional RT-PCR approach using benchtop qPCR instruments instead. Therefore, this DMF system can be applied to rapid contagious disease diagnosis in resource-limited settings, where centralized laboratories and skilled technicians are missing.

## Figures and Tables

**Table 1:** RT-qPCR primers and probes used in the study.

Spot	Target Region	Primer Source	Label	Sequence
1	Mycoplasma pneumoniae mgpA	designed in Primer Premier 5	MP-FP	AGAATCTACCCTTTAACAATAACCG
			MP-RP	CCGCTAAAACGAGTTCCCTA
			MP-P	FAM-TACCACGGATGGCAGTTGCTGG-BHQ1
2	Chlamydophila pneumoniae 23S ribosomal RNA genes	designed in Primer Premier 5	CP-FP	TATGACCCGGAGGTATCCG
			CP-RP	CGGGTGTCGCCTTATATGC
			CP-P	HRF550-TGGGGCAACCCGATAGACTAATAG-BHQ1
3	Streptococcus pneumoniae autolysin gene	designed in Primer Premier 5	SP-FP	CGAACTCTTACGCAATCTAGCA
			SP-RP	TTCGTGCAATACTCGTGCG
			SP-P	HRF550-CCAGCTAAACTCCCTGTATCAAGCG-BHQ1
4	Human respiratory syncytial virus A fusion protein	designed in Primer Premier 5	RSVA-FP	AAACAGATGTAAGCAGCTCCGT
			RSVA-RP	CGATTTTTATTGGATGCTGTACAT
			RSVA-P	FAM-ACATCTCTAGGAGCCATTGTGTCATG-BHQ1
5	Human adenovirus hexon protein	designed in Primer Premier 5	ADV-FP	CTCGGAGTACCTGAGTCCGG
			ADV-RP	CGTGGGATTTCTAAACTCATTTC
			ADV-P	HRF550-CGCCACAGACACCTACTTCAATCTG-BHQ1
6	Human coronavirus HKU1 open reading frames 1ab	designed in Primer Premier 5	HKU1-FP	CACATGGTGATAGATTTTATCGC
			HKU1-RP	ATAATAGCAACCGCCACACAT
			HKU1-P	HRF550-CTTGCGAATGAATGTGCTCAAGTTT-BHQ1
7	Human coronavirus 229E nucleocapsid protein	designed in Primer Premier 5	229E-FP	CAGAAAACGAAAGATTGCTTCA
			229E-RP	CAAGCAAAGGGCTATAAAGAGA
			229E-P	HRF550-ATGGCTACAGTCAAATGGGCTGATGC-BHQ1
8	Human metapneumovirus fusion protein	designed in Primer Premier 5	HMPV-FP	CTGTCAGCTTCAGTCARTTCAAC
			HMPV-RP	CAATGATATTGCYGGTGTTAT
			HMPV-P	FAM-TGTTGTGCGGCAGTTTTCAGACAAT-BHQ1
9	SARS-CoV-2 nucleocapsid protein	NHC of P.R. China, 2020	N-FP	GGGGAACTTCTCCTGCTAGAAT
			N-RP	CAGACATTTTGCTCTCAAGCTG
			N-P	FAM-TTGCTGCTGCTTGACAGATT-TAMRA
10	SARS-CoV-2 open reading frames 1ab	NHC of P.R. China, 2020	ORF1-FP	CCCTGTGGGTTTTACACTTAA
			ORF1-RP	ACGATTGTGCATCAGCTGA
			ORF1-P	FAM-CCGTCTGCGGTATGTGGAAAGGTTATGG-BHQ1
11	Influenza A virus matrix gene 1 & 2	Chinese National lnfluenza Center, 2017	FLUA-FP	GACCRATCCTGTCACCTCTGAC
			FLUA-RP	GGGCATTYTGGACAAAKCGTCTACG
			FLUA-P	FAM-TGCAGTCCTCGCTCACTGGGCACG-BHQ1
12	Influenza B virus hemagglutinin	Chinese National lnfluenza Center, 2017	FLUB-FP	AGACCAGAGGGAAACTATGCCC
			FLUB-RP	TCCGGATGTAACAGGTCTGACTT
			FLUB-P1	FAM-CAGACCAAAATGCACGGGGAAHATACC-BHQ1
			FLUB-P2	FAM-CAGRCCAATGTGTGTGGGGAYCACACC-BHQ1

**Table 2:** Final concentration of primers and probes used in the study.

Target	Primer F Final Concentration	Primer R Final Concentration	Primer P Final Concentration	Maximum RFI
MP	0.4 μM	0.4 μM	133 nM	1.6
CP	0.4 μM	0.4 μM	200 nM	1.6
SP	0.4 μM	0.4 μM	200 nM	1.5
RSVA	0.4 μM	0.4 μM	240 nM	1.8
ADV	0.4 μM	0.4 μM	400 nM	1.5
HKUI	0.4 μM	0.4 μM	200 nM	1.3
229E	0.4 μM	0.4 μM	160 nM	1.8
HMPV	0.4 μM	0.4 μM	100 nM	1.8
N	0.4 μM	0.4 μM	200 nM	1.6
ORF1ab	0.4 μM	0.4 μM	260 nM	1.8
FLUA	0.4 μM	0.4 μM	160 nM	1.7
FLUB	0.4 μM	0.4 μM	200 nM	1.5

Adjustments of final concentration of primers and probes are shown. The concentrations of forward and reverse primers are 0.4 μM and the concentration of probes is adjusted until the maximum RFI is about 1.3–1.8.

**Table 3:** Summary of sensitivity of off-chip RT-qPCR.

Type of Infection	Dynamic Range	Standard Curve	Rsq	Mean Ct	Detection Limit (Copies/T)
MP	1.5 × 10^1^ to 1.5 × 10^7^	y = −3.4256x + 41.514	0.995	22.50	15
CP	1.5 × 10^2^ to 1.5 × 10^7^	y = −3.4841x + 41.053	0.998	21.75	150
SP	1.5 × 10^2^ to 1.5 × 10^7^	y = −3.7385x + 42.311	0.998	22.06	150
RSV	1.5 × 10^1^ to 1.5 × 10^7^	y = −3.6142x + 41.853	0.999	21.95	15
ADV	1.5 × 10^1^ to 1.5 × 10^7^	y = −3.4086x + 41.313	0.989	22.51	15
HKUI	1.5 × 10^2^ to 1.5 × 10^7^	y = −3.4600x + 39.304	0.999	21.99	150
229E	1.5 × 10^2^ to 1.5 × 10^7^	y = −3.4672x + 39.896	0.998	22.36	150
HMPV	1.5 × 10^2^ to 1.5 × 10^7^	y = −3.4859x + 38.982	0.998	21.29	150
N	1.5 × 10^2^ to 1.5 × 10^7^	y = −3.5771x + 41.425	0.999	22.01	150
ORF1ab	1.5 × 10^2^ to 1.5 × 10^7^	y = −3.4684x + 41.344	0.998	22.30	150
FLUA	1.5 × 10^1^ to 1.5 × 10^7^	y = −3.4674x + 40.598	0.999	21.59	15
FLUB	1.5 × 10^2^ to 1.5 × 10^7^	y = −3.7135x + 43.109	0.999	23.04	150

**Table 4:** Summary of sensitivity of on-chip RT-qPCR.

Type of Infection	Dynamic Range	Standard Curve	Rsq	Mean Ct	Detection Limit (Copies/T)
MP	1.2 × 10^2^ to 1.2 × 10^6^	y = −3.5667x + 38.085	0.986	20.76	120
CP	1.2 × 10^2^ to 1.2 × 10^6^	y = −3.9103x + 40.054	0.984	21.13	120
SP	1.2 × 10^2^ to 1.2 × 10^6^	y = −3.5293x + 40.611	0.939	23.36	120
RSV	1.2 × 10^2^ to 1.2 × 10^6^	y = −3.7060x + 37.213	0.996	18.91	120
ADV	1.2 × 10^1^ to 1.2 × 10^6^	y = −3.6477x + 38.470	0.981	21.26	12
HKUI	1.2 × 10^1^ to 1.2 × 10^6^	y = −3.4883x + 38.630	0.971	21.63	12
229E	1.2 × 10^2^ to 1.2 × 10^6^	y = −3.7153x + 41.180	0.933	22.80	120
HMPV	1.2 × 10^2^ to 1.2 × 10^6^	y = −4.2580x + 44.229	0.977	23.32	120
N	1.2 × 10^2^ to 1.2 × 10^6^	y = −4.3620x + 42.910	0.994	21.15	120
ORF1ab	1.2 × 10^2^ to 1.2 × 10^6^	y = −3.9967x + 42.816	0.947	24.22	120
FLUA	1.2 × 10^2^ to 1.2 × 10^6^	y = −3.0850x + 40.021	0.958	25.34	120
FLUB	1.2 × 10^2^ to 1.2 × 10^6^	y = −3.6880x + 42.363	0.982	24.69	120

**Table 5:** Summary of testing results of 40 clinical samples by RT-qPCR in both off-chip and on-chip mode.

		RT-qPCR Off-Chip		
		Positive	Negative	Total
**RT-qPCR On-Chip**	**Positive**	28	0	28
	**Negative**	2	450	452
	**Total**	30	450	480

**Table 6:** Symmetric measures of RT-qPCR off-chip and on-chip on 40 clinical samples.

	Value	Asymp. Std. Error ^1^	Approx. T ^2^	Approx. Siq.
Measure of Agreement Kappa	0.963	0.026	21.119	0.000
N of Valid Cases	480			

^1^ The null hypothesis is not assumed. ^2^ The asymptotic standard error is used to assume the null hypothesis.

**Table 7:** Comparison of available diagnostic assays for respiratory pathogens.

Method		Multiple Detection	Within 2 h	High Sensitivity	High Specificity
Conventional diagnosis [26,27,28]	Bacterial culture	×	×	√	√
	Immunofluorescence	×	√	×	×
	ELISA	×	√	×	×
Molecular diagnostics [26,27,28]	DMF with LAMP	×	√	√	√
	DMF with RT-qPCR	√	√	√	√

## Data Availability

Not applicable.

## Supplementary Materials

The following supporting information can be downloaded at: https://www.mdpi.com/article/10.3390/mi13101650/s1, Table S1: Specificity of RT-qPCR on-chip in the study. Table S2: Test result of clinical samples using both off-chip and on-chip RT-qPCR methods.
