# Supplementary material for: A Digital Microfluidic RT-qPCR Platform for Multiple Detections of Respiratory Pathogens
Source: Micromachines (Basel). 2022 Sep 30;13(10):1650. doi: 10.3390/mi13101650 (PMC9611846; doi:10.3390/mi13101650)
Supplement: Supplementary file 1 [file micromachines-13-01650-s001.zip › micromachines-1906297-supplementary.pdf]

Supplementary information to

A digital microfluidic RT-qPCR platform for multiple detection of respiratory pathogens

Huitao Huang [1, †], Kaisong Huang [1, †], Yun Sun [2], Dasheng Luo [2], Min Wang [2], Tianlan Chen [2,3], Mingzhong Li [4], Junwei Duan [5], Liqun Huang[1] and Cheng Dong [6, *]

[1] Zhuhai Center for Disease Control and Prevention, Zhuhai 519087, China
[2] Digifluidic Biotech Ltd., Zhuhai, 519000, China
[3] Guangzhou Nansha IT Park Postdoctoral Programme, Guangzhou 511466, China
[4] State Key Laboratory of Analog and Mixed-Signal VLSI, University of Macau, Macao 999078, China
[5] College of Information Science and Technology, Jinan University, Guangzhou 510632, China
[6] School of Intelligent Systems Science and Engineering/JNU-Industry School of Artificial Intelligence, Jinan University, Zhuhai 519000, China
* Correspondence: dongcheng@jnu.edu.cn
† These authors contributed equally to this work.

1 Test result of Specificity of RT-qPCR on chip

The testing results of specificity of RT-qPCR on chip are listed as below in table S1. In this table, the column 'No.' refer to the No. Of the samples, '+' and '-' means positive and negative testing result respectively. HRV = human rhinovirus, KP = Klebsiella pneumonia, HI = Hemophilus influenza. No.1 to No.18 are negative and positive controls. No.19 to No.38 are blank control groups.

Table S1 Specificity of RT-qPCR on chip in the study.

No.	Target	Test result											
		MP	CP	SP	RSV	ADV	HKUI	229E	HMPV	N	ORF1ab	FLUA	FLUB
1	MP	+	-	-	-	-	-	-	-	-	-	-	-
2	CP	-	+	-	-	-	-	-	-	-	-	-	-
3	SP	-	-	+	-	-	-	-	-	-	-	-	-

4	RSV	-	-	-	+	-	-	-	-	-	-	-	-
5	ADV	-	-	-	-	+	-	-	-	-	-	-	-
6	HKUI	-	-	-	-	-	+	-	-	-	-	-	-
7	229E	-	-	-	-	-	-	+	-	-	-	-	-
8	HMPV	-	-	-	-	-	-	-	+	-	-	-	-
9	N	-	-	-	-	-	-	-	-	+	-	-	-
10	ORF1ab	-	-	-	-	-	-	-	-	-	+	-	-
11	FLUA	-	-	-	-	-	-	-	-	-	-	+	-
12	FLUB	-	-	-	-	-	-	-	-	-	-	-	+
13	SARS	-	-	-	-	-	-	-	-	-	-	-	-
14	MERS	-	-	-	-	-	-	-	-	-	-	-	-
15	PIV	-	-	-	-	-	-	-	-	-	-	-	-
16	HRV	-	-	-	-	-	-	-	-	-	-	-	-
17	KP	-	-	-	-	-	-	-	-	-	-	-	-
18	HI	-	-	-	-	-	-	-	-	-	-	-	-
19~38	Neg	-	-	-	-	-	-	-	-	-	-	-	-

2 Test result of clinical samples

In total, 40 clinical samples are tested in this study, using both on/off chip RT-qPCR methods. The testing results are listed as below in table S2. In this table, the column ‘No.’ refers to the No. Of the samples, ‘+’ and ‘-’ mean positive and negative testing result respectively.

Table S2 Test result of clinical samples using both off chip and on chip RT-qPCR methods

No.	Test result: on chip / off chip											
	MP	CP	SP	RSV	ADV	HKUI	229E	HMPV	N	ORF1ab	FLUA	FLUB
1	- / -	- / -	+ / +	- / -	- / -	- / -	- / -	+ / +	- / -	- / -	- / -	- / -
2	- / -	- / -	- / -	- / -	- / -	- / -	- / -	- / -	- / -	- / -	- / -	- / -
3	- / -	- / -	- / -	- / -	- / -	- / -	- / -	+ / +	- / -	- / -	- / -	- / -
4	- / -	- / -	- / -	+ / +	- / -	- / -	- / -	- / -	- / -	- / -	- / -	- / -
5	- / -	- / -	- / -	+ / +	- / -	- / -	- / -	- / -	- / -	- / -	- / -	- / -
6	- / -	- / -	- / -	- / -	+ / +	- / -	- / -	- / -	- / -	- / -	- / -	- / -
7	- / -	- / -	- / -	- / -	+ / +	- / -	- / -	- / -	- / -	- / -	- / -	- / -
8	- / -	- / -	- / -	- / -	+ / +	- / -	- / -	- / -	- / -	- / -	- / -	- / -
9	- / -	- / -	- / -	- / -	- / -	- / -	- / -	- / -	- / -	- / -	- / -	- / -
10	- / -	- / -	- / -	- / -	- / -	- / -	- / -	- / -	- / -	- / -	- / -	- / -
11	+ / +	- / -	- / -	- / -	- / -	- / -	- / -	- / -	- / -	- / -	- / -	- / -
12	+ / +	- / -	- / -	- / -	- / -	- / -	- / -	- / -	- / -	- / -	- / -	- / -
13	- / -	- / -	- / -	- / +	- / -	- / -	- / -	- / -	- / -	- / -	- / -	- / -
14	- / -	- / -	- / -	- / -	- / -	- / -	- / -	- / -	- / -	- / -	- / -	- / -
15	- / -	- / -	- / -	- / -	- / -	- / -	- / -	- / -	- / -	- / -	- / -	- / -
16	- / -	- / -	+ / +	- / -	- / -	- / -	- / -	- / -	- / -	- / -	- / -	- / -
17	- / -	- / -	- / -	- / -	- / -	- / -	- / -	- / -	- / -	- / -	- / -	- / -
18	- / +	- / -	- / -	- / -	- / -	- / -	- / -	- / -	- / -	- / -	- / -	- / -
19	- / -	- / -	- / -	- / -	- / -	- / -	- / -	- / -	- / -	- / -	- / -	- / -
20	- / -	- / -	- / -	- / -	- / -	- / -	- / -	- / -	- / -	- / -	- / -	- / -
21	- / -	- / -	- / -	- / -	- / -	- / -	- / -	- / -	- / -	- / -	- / -	+ / +
22	- / -	- / -	- / -	- / -	- / -	- / -	- / -	- / -	+ / +	+ / +	- / -	- / -
23	- / -	- / -	- / -	- / -	- / -	- / -	- / -	- / -	- / -	- / -	+ / +	- / -
24	- / -	- / -	- / -	- / -	- / -	- / -	- / -	- / -	- / -	- / -	- / -	+ / +
25	- / -	- / -	- / -	- / -	- / -	- / -	- / -	- / -	- / -	- / -	- / -	- / -
26	- / -	- / -	- / -	- / -	- / -	- / -	- / -	- / -	- / -	- / -	+ / +	- / -
27	- / -	- / -	- / -	- / -	- / -	- / -	- / -	- / -	- / -	- / -	- / -	- / -
28	- / -	- / -	- / -	- / -	- / -	- / -	- / -	- / -	- / -	- / -	- / -	+ / +
29	- / -	- / -	- / -	- / -	- / -	- / -	- / -	- / -	- / -	- / -	+ / +	- / -
30	- / -	- / -	- / -	- / -	- / -	- / -	- / -	- / -	- / -	- / -	- / -	- / -
31	- / -	- / -	- / -	- / -	- / -	- / -	- / -	- / -	- / -	- / -	- / -	+ / +
32	- / -	- / -	- / -	- / -	- / -	- / -	- / -	- / -	- / -	- / -	+ / +	- / -
33	- / -	- / -	- / -	- / -	- / -	- / -	- / -	- / -	- / -	- / -	- / -	- / -

34	- / -	- / -	- / -	- / -	- / -	- / -	- / -	- / -	- / -	- / -	+ / +	- / -
35	- / -	- / -	- / -	- / -	- / -	- / -	- / -	- / -	+ / +	+ / +	- / -	- / -
36	- / -	- / -	- / -	- / -	- / -	- / -	- / -	- / -	- / -	- / -	- / -	+ / +
37	- / -	- / -	- / -	- / -	- / -	- / -	- / -	- / -	- / -	- / -	- / -	- / -
38	- / -	- / -	- / -	- / -	- / -	- / -	- / -	- / -	- / -	- / -	+ / +	- / -
39	- / -	- / -	- / -	- / -	- / -	- / -	- / -	- / -	- / -	- / -	- / -	+ / +
40	- / -	- / -	- / -	- / -	- / -	- / -	- / -	- / -	- / -	- / -	+ / +	- / -
